# Examining the Relationship Between Speech Perception, Production Distinctness, and Production Variability

**DOI:** 10.3389/fnhum.2021.660948

**Published:** 2021-05-28

**Authors:** Hung-Shao Cheng, Caroline A. Niziolek, Adam Buchwald, Tara McAllister

**Affiliations:** ^1^Department of Communicative Sciences and Disorders, New York University, New York City, NY, United States; ^2^Department of Communication Sciences and Disorders, University of Wisconsin-Madison, Madison, WI, United States

**Keywords:** speech perception, speech production, production variability, speech motor control, individual differences

## Abstract

Several studies have demonstrated that individuals’ ability to perceive a speech sound contrast is related to the production of that contrast in their native language. The theoretical account for this relationship is that speech perception and production have a shared multimodal representation in relevant sensory spaces (e.g., auditory and somatosensory domains). This gives rise to a prediction that individuals with more narrowly defined targets will produce greater separation between contrasting sounds, as well as lower variability in the production of each sound. However, empirical studies that tested this hypothesis, particularly with regard to variability, have reported mixed outcomes. The current study investigates the relationship between perceptual ability and production ability, focusing on the auditory domain. We examined whether individuals’ categorical labeling consistency for the American English /ε/–/æ/ contrast, measured using a perceptual identification task, is related to distance between the centroids of vowel categories in acoustic space (i.e., vowel contrast distance) and to two measures of production variability: the overall distribution of repeated tokens for the vowels (i.e., area of the ellipse) and the proportional within-trial decrease in variability as defined as the magnitude of self-correction to the initial acoustic variation of each token (i.e., centering ratio). No significant associations were found between categorical labeling consistency and vowel contrast distance, between categorical labeling consistency and area of the ellipse, or between categorical labeling consistency and centering ratio. These null results suggest that the perception-production relation may not be as robust as suggested by a widely adopted theoretical framing in terms of the size of auditory target regions. However, the present results may also be attributable to choices in implementation (e.g., the use of model talkers instead of continua derived from the participants’ own productions) that should be subject to further investigation.

## Introduction

While speech perception and production are often studied separately, there is a sizable body of research establishing that these two domains are interdependent. Specifically, previous studies have suggested that an individual’s ability to perceive a sound contrast in their native language is related to the precision with which they produce the contrast. This relationship has been examined for different types of contrast, including vowels (Perkell et al., 2004a, 2008; Franken et al., 2017) and sibilants (Perkell et al., 2004b; Ghosh et al., 2010; Brunner et al., 2011). A theoretical account for this observed perception-production relationship is that both modalities share a phonetic target at some level of representation. While speech targets are presumed to have a multimodal representation with both auditory and somatosensory components, the focus in the present paper is on the auditory-perceptual domain. This gives rise to the hypothesis that individuals with more narrowly defined auditory targets should both perform better on perceptual tasks and produce speech sound contrasts more distinctly, e.g., with greater separation between category means and less variability within each category (Perkell, 2012). However, previous empirical studies that directly tested this hypothesis have reported mixed results, especially regarding the relationship between perception ability and production variability (Perkell et al., 2008; Franken et al., 2017). We discuss these inconsistencies in detail below after a brief review of the broader literature.

One possible reason for the inconsistent findings that form the focus of this paper is that there are no agreed-upon methods to measure the size of the auditory target at the representational level. For example, within the aforementioned studies, different types of discrimination task were used to measure individuals’ ability to detect a difference in a pair of sounds. These included an ABX task (Perkell et al., 2004a,b), where participants are asked to determine whether the third sound (i.e., X) is the same as the first (i.e., A) or the second sound (i.e., B), and a 4-interval 2-alternative forced-choice discrimination task (Perkell et al., 2008; Franken et al., 2017), where participants are asked to decide whether the second or the third sound is different from the rest. In addition to discrimination tasks, other studies examining perception-production relations have used an identification task, where participants are asked to partition points along an acoustic continuum into distinct phonemic categories (McAllister Byun and Tiede, 2017; Park et al., 2019). While the terms *auditory acuity* or *auditory-perceptual acuity* have been used to refer to individuals’ perceptual ability across these different tasks in previous published literature, it is important to recall that these tasks measure distinct aspects of perception ability. Therefore, it may be helpful to review what specific abilities are measured by each perception task before investigating the relationship between perception and production.

### Differences in Perception Tasks

Two major types of task are commonly used to study speech perception: identification tasks and discrimination tasks. In identification tasks, listeners explicitly label a sound as belonging to one category or another, whereas in a discrimination task, listeners hear two sounds and have to respond whether they are the same or different (also called AX tasks). In its original formulation, the concept of *categorical perception* was defined as a combination of participants’ behavior in each of these tasks (Liberman et al., 1967). In an identification task, participants are asked to provide a label for sounds that are equally spaced along on a continuum and for native contrasts, there is a boundary where sounds on one side are labeled differently than the other side. A contrast is perceived categorically if discrimination among different sounds on the continuum that share a label is poor, whereas discrimination among sounds that are equally-spaced on the continuum but have different labels is strong. While consonants tend to be perceived categorically, with discrimination ability following the identification boundary (Liberman et al., 1967), early studies noted that vowel discrimination did not seem to be categorical and that participants can distinguish vowels even when they identify them with the same label (Studdert-Kennedy et al., 1972; Pisoni, 1973, 1975). However, changing aspects of the discrimination task (such as lengthening the inter-stimulus interval between items) can cause listeners to exhibit a more categorical pattern with discrimination following the labeling boundary (Pisoni, 1973). This suggests that, in terms of vowel discrimination, listeners may use different modes of perception for vowel stimuli: a categorical mode in which listeners map the input onto abstract phonemic categories and using those labels to inform their response (Pisoni, 1973, 1975), and an auditory mode in which listeners attend and respond to continuous phonetic detail (Pisoni, 1973, 1975; Gerrits and Schouten, 2004).

Previous studies have suggested that the varying the structure of the perception task can influence listeners’ use of different modes of perception with respect to vowel discrimination (Pisoni, 1973, 1975; Gerrits and Schouten, 2004). Pisoni (1975) compared participants’ discrimination of the American English/i/–/I/contrast in an ABX task to their performance in a 4IAX (e.g., A-A, A-B) task. In the 4IAX task, participants were asked to determine whether the first (e.g., A-A) or the second (e.g., A-B) pair of sounds contain the same stimulus. They found that participants exhibited performance more typical of categorical perception in the ABX task than in the 4IAX task. That is, they showed higher discrimination accuracy between than within vowel categories, although their within-category discrimination accuracy was also above chance. In contrast, in the 4IAX task, participants exhibited high discrimination accuracy, regardless of whether a pair of sounds were taken from within or between categories. The results suggest that participants may use both categorical and auditory information to make a discrimination judgment in an ABX task, whereas they rely primarily on auditory information in the 4IAX task. Similarly, in their study examining Dutch/u/–/i/contrast, Gerrits and Schouten (2004) found that participants’ discrimination performance obtained from a 4-interval 2-alternative forced-choice task did not exhibit the asymmetry typical of categorical perception. This suggests that the 4-interval 2-alternative forced-choice discrimination task, like the 4IAX task, can be performed using only auditory information, with no labeling process. In sum, these results suggest that vowel discrimination can be either a categorical or an auditory task and that different discrimination tasks tap into different levels of processing. We propose a terminological clarification in connection with this distinction. In previous studies of the perception-production relationship (Perkell et al., 2004a,b, 2008; Franken et al., 2017), the term *auditory acuity* has been widely adopted to describe performance on listening tasks, regardless of the nature of the discrimination task used. Because of the differences between these tasks, here we use the term *ABX discrimination thresholds* to specifically refer to individuals’ discrimination ability measured from an ABX task, and *4I2AFC discrimination thresholds* to refer to individuals’ discrimination ability measured from a 4-interval 2-alternative forced-choice task. It should be further noted that previous studies that examined perception-production relationships commonly used an adaptive staircase procedure to measure the smallest difference (i.e., just noticeable difference) a listener can discriminate for a given contrast (Perkell et al., 2008; Ghosh et al., 2010; Franken et al., 2017).

On the other hand, identification tasks clearly tap into the categorical mode of perception, as listeners are required to respond by classifying a stimulus into a phonemic category. However, listeners’ sensitivity to within-category detail can influence the consistency with which they make these classifications, and therefore auditory perception may also be relevant to performance on an identification task. In a typical identification task, participants’ responses along a synthesized continuum are plotted and fitted with a sigmoid function. This fitted function can be used to find the location of the boundary between categories (typically the point on the sigmoid representing equal probability of both categories), as well as the width of the boundary region (e.g., the distance from the 25th to the 75th percentile of probability along the fitted function), which is inversely related to the slope of the sigmoid function. The width of the boundary region is driven primarily by the consistency with which listeners partition the ambiguous points around the boundary into each category, with a smaller boundary width representing higher categorical labeling consistency (McAllister Byun and Tiede, 2017; Park et al., 2019). Some previous studies (McAllister Byun and Tiede, 2017; Cialdella et al., 2020) have used the term *perceptual acuity* or *auditory-perceptual acuity* to refer to this measure of boundary width; however, here we will adopt the term *categorical labeling consistency* as a better match for the nature of the identification task.

### Links Between Speech Perception and Production Distinctness

As noted above, multiple previous studies have suggested that individuals’ ability to discriminate a sound contrast is reflected in their production of the contrast. For example, Perkell et al. (2004a) examined perception and production of the vowel contrasts /ɑ/–/ʌ/and/u/–/ʊ/ in American English. An ABX task was used to measure participants’ discrimination ability within and between vowel categories. A synthesized continuum for each vowel contrast was created using natural tokens produced by two model speakers. Participants were dichotomized into high and low perceptual ability groups based on their between-category ABX discrimination thresholds. Production ability was quantified as the Euclidean distance between the centroids of the two vowels in each contrast. They found that participants who were in the high perceptual ability group produced the vowel contrasts with greater separation (i.e., larger contrast distance) in both acoustic and articulatory space than those in the low group. Perkell et al. (2004b) reported similar findings for the contrast between the sibilants/s/–/∫/ in American English (as did Ghosh et al., 2010).^1^ These results suggested that individuals who were better at discriminating sounds at the category boundary also produced the contrast with more distinction.

Another line of research has suggested that the production distinctness of sound categories is related to individuals’ perceptual ability for the contrast as measured with an identification task. For example, McAllister Byun and Tiede (2017) examined the relationship between perception and production of American English /ɹ/ in typically developing children. They found that children with higher categorical labeling consistency in identifying sounds along a continuum from /ɹ/to/w/produced the /ɹ/ sound with higher degree of rhoticity (i.e., a smaller distance between F2 and F3). Whereas the aforementioned contrasts are phonologically contrastive in American English, Park et al. (2019) examined the perception-production relationship in a different context, examining voice quality with respect to breathy phonation and modal phonation. The results of their study showed that speakers with higher categorical labeling consistency in classifying tokens into breathy and modal phonation categories also produced sounds with less breathiness. These findings suggest that individuals’ performance in identifying stimuli around the categorical boundary is also linked to the distinctness of their production of one target category with respect to the acoustic dimension that is relevant to the contrast. However, it is important to note that both of these studies measured only one category. To the best of our knowledge, no study has specifically examined the extent to which categorical labeling consistency relates to a production measure that examines the separation between both categories (e.g., vowel contrast distance).

### Links Between Speech Perception and Production Variability

When measuring individuals’ ability to produce a given sound contrast, most studies have focused on the difference between sounds, defined in terms of either mean acoustic characteristics (Perkell et al., 2004a,b; Ghosh et al., 2010) or mean kinematic properties (Perkell et al., 2004a). However, averaging over repeated trials omits information about trial-to-trial variability, which may be of relevance to both perception and production. In fact, Chao et al. (2019) found that the location of participants’ perceptual boundary between /ε/–/æ/ in American English, derived using an identification task, was correlated with the location of the boundary between these categories in production space, derived based on the distribution of tokens across repeated productions. That is, the categorical boundary was further away from the more variable vowel in the contrast. Their results suggest that production variability is not simply a reflection of motoric noise in the production system but reflects the organization of sound categories in the representation of both perception and production. In addition, as mentioned above, if a shared representation underlies both perception and production, individuals with a more narrowly defined auditory target could be expected to exhibit both higher perception ability and lower production variability.

Previous studies that empirically tested this particular hypothesis, however, have reported mixed results. For example, Perkell et al. (2008) measured participants’ 4I2AFC discrimination thresholds (i.e., just noticeable difference in a 4-interval 2-alternative forced-choice task) in differentiating acoustic differences at their categorical boundary for the vowel contrasts/I/–/ε/ and /ε/–/æ/. Production variability was quantified as the area of ellipses representing 95% confidence intervals around the acoustic values (i.e., F1 and F2) of each target. They found that participants with smaller 4I2AFC discrimination thresholds exhibited lower production variability across the two vowels (i.e., smaller area of the ellipse). However, this relationship was less clear in a study by Franken et al. (2017), which used similar methodology to that of Perkell et al. (2008). While the results of their study showed that participants who had smaller 4I2AFC discrimination thresholds in discriminating between Dutch /ε/–/I/ and /ɑ/–/ɔ/ also produced the vowels with lower trial-to-trial variability, this relationship was only found when the analysis assessed variability in Mel-frequency cepstrum coefficients (MFCC). Another analysis using measurements of formant frequencies on the Bark scale failed to show a significant association. One possible explanation for the equivocal results across these studies is their use of the 4-interval 2-alternative forced-choice discrimination task. As stated previously, participants rely mostly on low-level auditory information to perform discrimination in this type of task. However, the notion of “narrowness of the auditory target” can be expected to have some relation to how listeners classify tokens in the vicinity of phonetic category boundaries (e.g., whether they have a strict or lenient cutoff for category inclusion). Thus, it may not be optimal to use a task that taps into low-level auditory perception in this context. It is possible that stronger associations between perception and production variability could be observed using perception tasks that can measure how consistently participants categorize an acoustic continuum into either of the sound categories in a given contrast, such as an identification task (McAllister Byun and Tiede, 2017; Park et al., 2019).

While the authors of the above studies measured trial-to-trial production variability using a single timepoint (e.g., acoustic values obtained at the midpoint of each target sound), other studies have used a different measure that examines changes in variability over time within each utterance (Niziolek et al., 2013; Bakst and Niziolek, 2019). This type of analysis of production variability takes the mechanism of auditory feedback control into consideration. Although numerous different models of speech-motor control have been proposed, auditory feedback is consistently identified as an important factor that speakers use to modify their ongoing speech production. Specifically, speakers adjust their speech output when the auditory feedback they receive deviates from an internal prediction of auditory feedback (for a review, see Parrell et al., 2019). Based on this control mechanism, Niziolek et al. (2013) found that speakers exhibited an online corrective behavior, such that productions that initially fell on the periphery of a speaker’s distribution of productions for a given vowel tended to move closer to the center of the region by the midpoint of the vowel. When looking across multiple trials, this behavior, termed centering, is hypothesized to indirectly reflect the size of the auditory target in the speaker’s stored representation because the correction was undertaken when the speaker’s auditory feedback indicated that the form produced deviated from the intended target. That is, for speakers who have the same initial variability, speakers with a narrower auditory target are predicted to exhibit a greater magnitude of centering than speakers with a wider target region. It is worth noting that this analysis is sensitive to the magnitude of initial variability because the amount of centering is bounded by how variable the production is at vowel onset. Thus, there can be a ceiling effect on the amount of centering, especially in individuals with a small magnitude of initial variability, because such speakers have a limited amount of space for correction.

Indirect evidence for a relationship between an individual’s perception ability and their magnitude of centering was found in a recent study comparing the amount of centering in the production of native versus non-native sounds (Bakst and Niziolek, 2019). In this study, the authors compared initial production variability and the magnitude of centering in American English vowels to those in French vowels in native speakers of American English who had at least intermediate-level knowledge of French. They found that participants produced higher initial variability in French vowels than in English vowels. However, a higher amount of centering was found in English vowels than in French vowels. This result provides a suggestion that amount of centering is related to perceptual ability, since native speakers have a better-defined auditory target for their native-language vowels (English) than for L2 vowels (French). However, no previous studies have directly examined the relationship between an individual’s perception ability and the magnitude of centering in production within a single language context.

### Current Study

The present study aims to address questions left unanswered by the previous literature reviewed above. This study will examine the relationship between perception and production of the American English /ε/–/æ/ contrast using data from a previous study (Klaus et al., 2019). Specifically, we will investigate the extent to which individuals’ categorical labeling consistency in an identification task relates to production distinctness of the vowel contrast, as well as the variability of their production of each vowel. Furthermore, both of the aforementioned measures related to production variability (i.e., area of the ellipse around productions in auditory-acoustic space and centering) will be examined in association with the perceptual measure. The overarching theoretical hypothesis was that individuals with more narrowly defined auditory targets at a representational level would be expected to show a more consistent partitioning of ambiguous tokens around the boundary, as well as more precise production of the contrast, than listeners with broadly specified or overlapping target regions. Thus, individuals with higher categorical labeling consistency (i.e., smaller boundary width) would be expected to also produce the contrast more distinctly (i.e., larger vowel contrast distance). In terms of production variability, we hypothesized that individuals with higher categorical labeling consistency would produce the two vowels with less variability as measured in terms of area of the ellipse. Given that the extent to which centering relates to perceptual measures remains incompletely understood, we considered two possibilities regarding the relationship between categorical labeling consistency and the magnitude of centering. If the self-correction process is driven primarily by the overall narrowness of the auditory targets for the two vowels, we would expect individuals’ categorical labeling consistency to be related to the magnitude of centering. That is, individuals with higher categorization consistency for the two vowels would be expected to exhibit a larger magnitude of centering. However, it should be noted that this relationship might be obscured by differences in variability across participants that are larger than the differences in narrowness of auditory targets. On the other hand, if the ability to detect subtle acoustic differences within acoustic categories is most important to the centering process, we would not expect individuals’ categorical labeling consistency to be related to the magnitude of centering, because categorical labeling consistency does not measure individuals’ ability to detect subtle within-category acoustic differences.

## Materials and Methods

### Participants

The data for the present study was drawn from Klaus et al. (2019). A total of 37 female participants ranging in age from 18 to 33 years (mean = 22.25 yr, SD = 3.56 yr) were recruited and completed the original study. An additional three participants were initially consented and participated in the study; however, they were excluded due to technical issues. The participants were all native speakers of American English and had reported no prior history of speech or hearing impairments. All participants passed a hearing screening at 1,000, 2,000, and 4,000 Hz at 20 dB hearing level (HL).

### Procedure

The perception and production data were taken from Klaus et al. (2019), which investigated the effects of perceptual training on participants’ performance in matching an explicitly presented visual-acoustic target. While the original experiment consisted of both perceptual training and speech motor learning components, only the perception and production data collected at the baseline time point (see below) were used for the current analyses. Below we describe the experimental procedures that were relevant to the current study.

### Baseline Probe

Participants were asked to repeat the words “head” and “had” 45 times each in random order. Immediately following this baseline production probe, participants were given a two-alternative forced-choice perceptual identification task where they had to classify a series of speech tokens as either “head” or “had.” Tokens were taken from a 11-step synthesized continuum from “head” to “had.” The continuum was created using the F1 and F2 values of a naturally produced token of “head” elicited from a native female speaker of American English. The F1 and F2 values were first modified using STRAIGHT synthesis (Kawahara et al., 2013) to create 41 equally-spaced continuum steps toward the formant values of the naturally produced “had” token from the same speaker. The vowel duration in each token was held constant. Eleven steps were selected from the full continuum in a way that oversampled the region surrounding the likely categorical boundary (i.e., the middle 50% of the full continuum length). That is, steps 3–9 on the continuum were more tightly spaced in their formant frequencies (mean distance = 26.4 Hz, SD = 10.3 Hz) than the tokens near the endpoints (mean distance = 40.2 Hz, SD = 15 Hz). For full details of the process, see Klaus et al. (2019). The eleven tokens along the continuum were presented 20 times each in random order to all participants for identification via mouse click (210 trials total). The same production and perception probes were administered again after the end of the training task, but the results from the post-test probes were not analyzed in the current study.

### Data Analysis

#### Categorical Labeling Consistency

**FIGURE 1:**
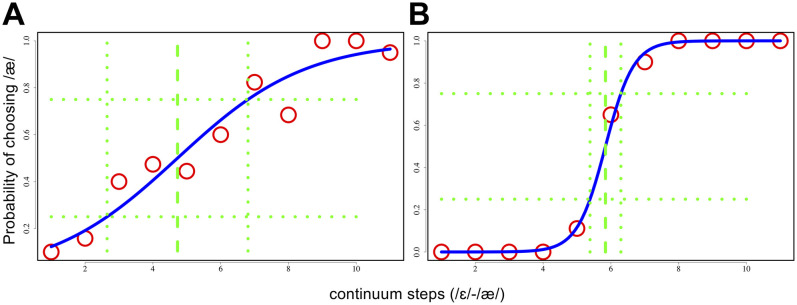
Examples illustrating the categorical labeling consistency measure. **(A)** A participant with lower consistency. **(B)** A participant with higher consistency. The center dashed line denotes the categorical boundary. The dotted line to the left of the boundary represents 25% probability. The line to the right of the boundary represents 75% probability.

Data from the perceptual identification task was used to calculate categorical labeling consistency for each participant. First the percentage of “head” responses at each of the synthetic continuum steps was plotted and fitted to a logistic function using a custom R script adopted from McAllister Byun and Tiede (2017) in RStudio (RStudio Team, 2020). The perceptual boundary between /ε/ and /æ/ was identified as the point on the continuum where the fitted logistic function reached its 50% probability point. Following McAllister Byun and Tiede (2017) and Park et al. (2019), the width of the boundary region, operationalized as the difference in continuum steps between the points where the fitted logistic function reached 25 and 75% probability, was used as an index of categorical labeling consistency. The width of the boundary region thus calculated reflects the steepness of the slope of the fitted logistic function, where a larger width indicates that the listener is less consistent in identifying intermediate tokens as /ε/ or /æ/ and a smaller width indicates that the listener makes a consistent distinction between the categories, as seen in Figure 1.

#### Formant Tracking and Extraction

The formant values of each participant’s baseline “head” and “had” utterance were analyzed in the following steps. Each utterance was uploaded to the DARLA web interface (Reddy and Stanford, 2015) that uses FAVE-extract (Rosenfelder et al., 2014) and the Montreal Forced Aligner (McAuliffe et al., 2017) for segmentation. We then visually inspected the alignment and manually corrected for any inaccuracies in segmentation. The vowel portion of each utterance was extracted and saved as an individual wav file using Praat (Boersma and Weenink, 2019). Vowel onset was defined as the beginning of the F2 trajectory associated with the onset of periodicity in the waveform. Vowel offset was defined as the end of the F2 trajectory associated with the offset of periodicity in the waveform. The formant values of each utterance were analyzed using the wave_viewer software package (Niziolek and Houde, 2015) in Matlab (The MathWorks Inc, 2019). The formants were tracked using linear predictive coding (LPC) analysis. The pre-emphasis frequency was set to 50 Hz for all vowels and all participants. To ensure stable formant tracking, we selected the window frame length on a per-participant basis. Either a 36-ms Hann window with a step size of 3 ms or an 18-ms Hann window with a step of 1.5 ms was used. The filter order was chosen on a per-participant and per-vowel basis. The formant estimation of each token was visually inspected and tokens with formant tracking errors were removed from the subsequent analysis. A total of 6 /ε/ tokens and a total of 11 /æ/ tokens from different participants were excluded from the analysis because of poor formant tracking quality. Formant values were estimated in Hz and then converted to the mel scale. Two time windows of interest were the initial portion (0 to 50 ms) and the midpoint (middle 50% relative to the total duration) of the vowel. Average formant values were then computed within each of the time windows for each utterance. The formant values associated with these two time windows were used to compute different measures of production ability, as described below. While the overall average duration for /ε/ and /æ/ and was 179 ms and 237 ms respectively, there were 6 participants whose average duration for /ϵ/ utterances were shorter than 150 ms, suggesting there was a potential overlap between the two time windows. Given the small number of tokens affected, we consider these instances of overlap unlikely to impact the interpretability of the centering analyses reported here.

#### Vowel Contrast Distance

To measure the degree of separation in each participant’s production of the /ε/–/æ/ contrast, we first computed the average midpoint F1 and F2 values across all token on a per-vowel and per-participant basis. Vowel contrast distance was then calculated as the Euclidean distance between the average F1 and F2 values of the two vowels for each participant (Perkell et al., 2004a, 2008). The distance value in the mel scale was then log-transformed.

#### Area of the Ellipse

**FIGURE 2:**
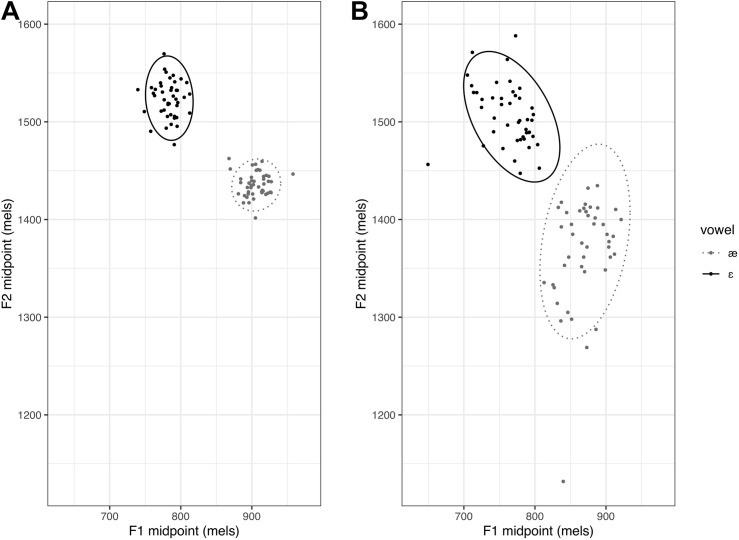
Examples illustrating area of the ellipse. Panel **(A)** represents a participant with small average area of the ellipse and panel **(B)** represents a participant with large average area of the ellipse.

To examine each participant’s overall production variability across repeated utterances of the two vowels, the midpoint F1 and F2 values of each token were used to compute an area of the ellipse measure for each vowel. Following Li et al. (2019), trial-to-trial production variability was quantified as the area of an ellipse representing the 95% confidence interval around the multivariate mean of the distribution of F1 and F2 values. Area of the ellipse was calculated first for each vowel separately. An average of these two areas of the ellipse was computed for each participant, representing the overall trial-to-trial production variability (Franken et al., 2017). The average area of the ellipse value was log-transformed. The participants with a large average area of the ellipse were considered to have higher trial-to-trial production variability than those with a smaller area of the ellipse, as shown in Figure 2.

#### Centering Ratio

**FIGURE 3:**
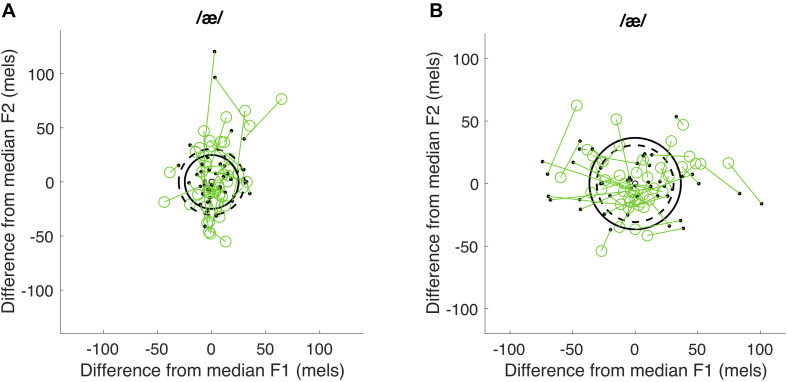
Examples illustrating centering ratio. Panel **(A)** shows a positive centering ratio and panel **(B)** a negative centering ratio. The F1 and F2 values were plotted as the difference from the median F1 and F2 values for each time window. The green open circles represent the difference between the initial F1 and F2 values for each utterance and the values representing the median values for each formant. The black dots represents the difference between the midpoint F1 and F2 values for each token and the median midpoint F1 and F2 values. Dashed line represents the average initial distance to the median and solid line represents the average midpoint distance to the median.

As described above, centering is intended to examine changes in production variability across time within each utterance. Centering was calculated using formant frequencies averaged across the initial and midpoint time windows, defined above. The median of these average F1 and F2 frequencies across repeated utterances was computed for each participant, vowel, and time window. For each utterance, the initial distance (d_*init*_) was calculated as the Euclidean distance between the initial F1 and F2 values for the utterance to the values representing the median initial distance for each formant (i.e., $d_{i\,n\,i\,t}=\sqrt{{\left(F\,1_{i\,n\,i\,t}-m\,e\,d\,i\,a\,n\,\left(F\,1_{i\,n\,i\,t}\right)\right)}^{2}+{\left(F\,2_{i\,n\,i\,t}-m\,e\,d\,i\,a\,n\,\left(F\,2_{i\,n\,i\,t}\right)\right)}^{2}}$). The midpoint distance was calculated as the Euclidean distance between the midpoint F1 and F2 values for a given token to the median midpoint F1 and F2 values (i.e., $d_{m\,i\,d}=\sqrt{{\left(F\,1_{m\,i\,d}-m\,e\,d\,i\,a\,n\,\left(F\,1_{m\,i\,d}\right)\right)}^{2}+{\left(F\,2_{m\,i\,d}-m\,e\,d\,i\,a\,n\,\left(F\,2_{m\,i\,d}\right)\right)}^{2}}$). Centering for each utterance was thus calculated as the change from the initial distance to the midpoint distance (i.e., d_*init*_ − d_*mid*_). A positive centering value suggests that the vowel formants began further away from the median but were corrected to be closer to the median at midpoint, whereas a negative centering ratio suggests that vowel formants tended to be further from the median at midpoint than at onset. This part of the analysis was performed using custom Matlab scripts from Niziolek and Kiran (2018). The mean centering value across all utterances was computed for each vowel and each participant, representing the average change in production variability from the initial to the midpoint time window. Because the possible amount of centering is bounded by participants’ level of variability at vowel onset (with lower variability leaving less room for correction), we normalized the average centering value of each vowel by the average initial distance of observed formant frequencies from the medians for that vowel. This normalized measure is termed the centering ratio. The average centering ratio across the two vowels was calculated and served as the final centering ratio measure. This part of the analysis was performed using R (Core Team R, 2020) in RStudio (RStudio Team, 2020). As shown in Figure 3, participants with a positive centering ratio were considered to exhibit an overall greater degree of corrective behavior than those with a near-zero or negative centering ratio.

## Results

### Data Cleaning

Prior to data cleaning, Shapiro-Wilk normality tests were used to test the normality assumption for each variable. The results of the tests revealed that categorical labeling consistency (*p* = 0.001) was not normally distributed, whereas vowel contrast distance (*p* = 0.1), area of the ellipse (*p* = 0.35), and centering ratio were (*p* = 0.81). Because not all variables were normally distributed, we chose to remove outliers using median absolute deviation (MAD) rather than standard deviation. For each variable, we compared each participant’s averaged results to the group median. Two participants were found to have a vowel contrast distance that fell two MADs away from the group median vowel contrast distance. Two participants were found to have an area of the ellipse that fell two MADs away from the group median area of the ellipse. Two participants were found to have a boundary width score that fell two MADs away from the group median boundary width. We removed these 6 participants from the following analyses. A total of 31 participants were included.

### Descriptive Statistics

In this section, we report summary statistics for each of the variables (i.e., categorical labeling consistency, vowel contrast distance, area of the ellipse, and centering ratio). Table 1 shows the mean, SD, and range for each of the measures. All values were derived from acoustic measures in the Mel scale except for categorical labeling consistency. The measures of vowel contrast distance and area of the ellipse appear to be fairly stable across participants: the standard deviation of each is small relative to the mean. Centering ratio appeared more variable, with some participants exhibiting negative average centering values and others exhibiting positive values. Finally, there was reasonable variability across participants in the measure of categorical labeling consistency, with a standard deviation of 0.56 continuum steps (out of a total of 11 steps).^2^ No participant exhibited a boundary width of 0, which would represent ceiling-level performance.^3^

**TABLE 1:** Summary of descriptive statistics (i.e., mean, standard deviation, minimum and maximum values) for each of the measures (*n* = 31).

	**Mean**	**SD**	**Min**	**Max**
categorical labeling consistency (continuum steps)	1.89	0.56	0.91	3.14
vowel contrast distance (mels, log-transformed)	5.11	0.20	4.62	5.48
area of the ellipse (mels, log-transformed)	8.90	0.36	8.19	9.64
centering ratio (mels)	0.06	0.10	−0.14	0.25

### Perception-Production Relationship

**FIGURE 4:**
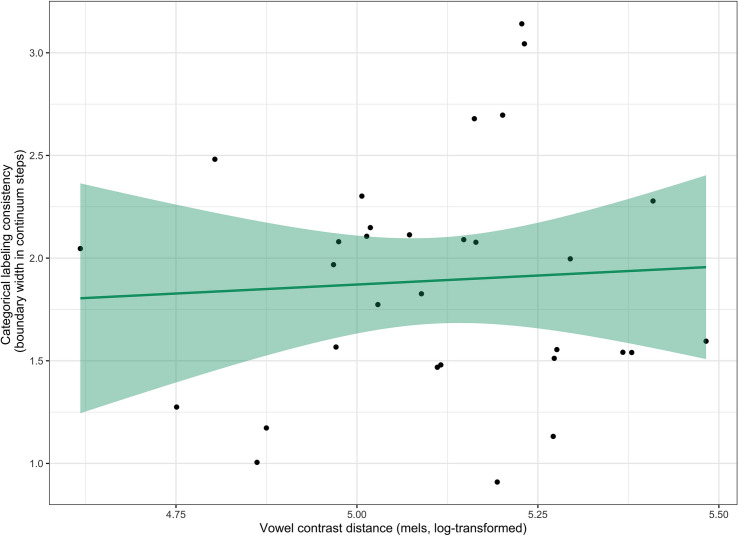
The correlation between categorical labeling consistency and vowel contrast distance. Shaded band represents a 95% confidence interval around the best-fit line.

Spearman’s correlation was used to examine whether categorical labeling consistency was correlated with vowel contrast distance. As seen in Figure 4, there was not a significant correlation between these two measures (ρ(29) = 0.03, *p* = 0.88).

**FIGURE 5:**
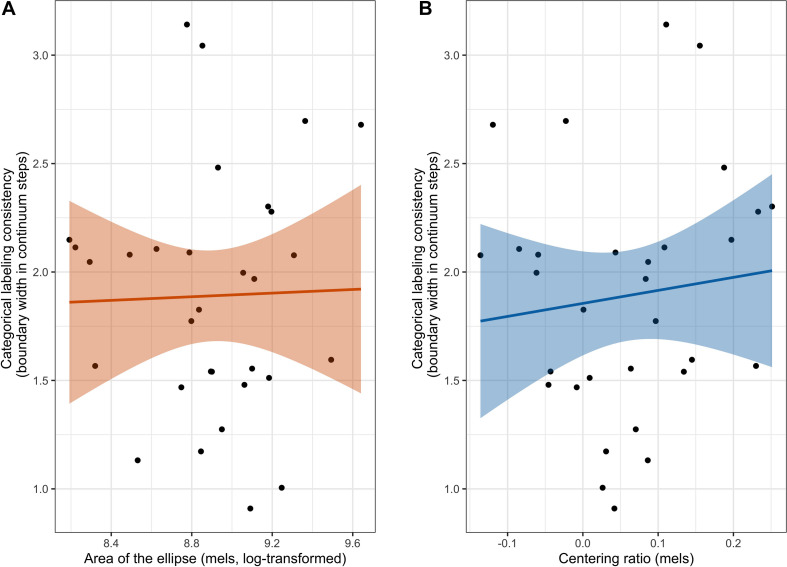
The correlation between categorical labeling consistency and both measures of production variability. The left figure **(A)** shows the correlation between categorical labeling consistency and area of the ellipse and the right figure **(B)** shows the correlation between categorical labeling consistency and centering ratio. Shaded band represents a 95% confidence interval around the best-fit line.

Spearman’s correlation was also used to examine whether categorical labeling consistency was correlated with either area of the ellipse or centering ratio. As seen in Figure 5, there was not a significant correlation between categorical labeling consistency and area of the ellipse (ρ(29) = −0.02, *p* = 0.91), nor was there a significant correlation between categorical labeling consistency and centering ratio (ρ(29) = 0.21, *p* = 0.26).

## Discussion

The present study investigated the perception-production relationship in an experimental task involving the American English /ε/–/æ/ contrast. A great deal of previous literature has endorsed a theoretical framing in which both perception and production are driven by the same target representations, and therefore perceptual ability should be correlated with both degree of separation between categories and within-category variability for the relevant contrast. However, empirical findings on the subject have been somewhat mixed, particularly on the topic of within-category variability (Perkell et al., 2008; Franken et al., 2017). Here we hypothesized that an association between perceptual ability and production variability might be more readily observed when perception ability was measured using categorical labeling consistency from a perceptual identification task. We examined this perceptual measure in association with vowel contrast distance (i.e., separation between the means of the two vowel categories), as well as two measures of production variability. The area of an ellipse representing a 95% confidence interval around the mean acoustic characteristics of a participant’s productions was used to quantify dispersion in acoustic space across repeated vowel utterances. A centering ratio measure was used to examine the magnitude of self-corrective behavior from onset to midpoint of each vowel produced. In contrast to our hypotheses, categorical labeling consistency was not found to significantly correlate with vowel contrast distance, nor with area of the ellipse. In addition, categorical labeling consistency was not found to significantly correlate with centering ratio.

### Relationship Between Perception Ability, Production Contrast Distinctness, and Production Variability

To our surprise, we did not observe an association between the consistency with which participants classified tokens along a synthetic continuum into /ε/ and /æ/ categories and vowel contrast distance in production. This ran counter to our expectations based on findings such as Perkell et al. (2004a), where individuals with smaller ABX discrimination thresholds for a vowel contrast also produced that contrast more distinctly than individuals with larger ABX discrimination thresholds. Of course, the two studies used different tasks (i.e., an identification task versus an ABX discrimination task), and as discussed throughout this paper, task differences may influence our ability to observe perception-production relations. However, we think this is not likely to be the explanation for two reasons. First, both studies measured participants’ perception ability around the categorical boundary regions, and the synthesized continua contained similar numbers of steps. Second, both identification and ABX discrimination tasks are thought to evoke a more categorical percept, and we thus expect both perception tasks to provide an indirect metric for the narrowness of a listener’s auditory target. Given these similarities, the difference in task might not be the best candidate to account for the discrepancy between the findings of the two studies. Another methodological difference that should be noted is that Perkell et al. (2004a) dichotomized their participants into two groups given that they observed a ceiling effect in participants’ between-category discrimination accuracy. While it is possible that the 11-step continuum used here may not be sufficiently fine-grained to reflect subtle individual differences in categorical labeling consistency, no participant in the present study achieved a boundary width of 0, which would represent ceiling-level performance. Instead, there was substantial individual variability in boundary width values, which should allow us to examine the relationship between categorical labeling consistency and vowel contrast distance in production as continuous measures. Thus, we did not dichotomize participants into groups.

With respect to the relationship between categorical labeling consistency and the area of the ellipse measure, the current findings are partially aligned with the results of previous studies. While Perkell et al. (2008) found that participants who showed higher sensitivity in discriminating vowel contrasts also produced the vowels with lower trial-to-trial variability, Franken et al. (2017) only observed this relationship in one of two analyses conducted. Above we speculated that the inconsistency of findings between Perkell et al. (2008) and Franken et al. (2017) might be attributable to their use of a 4-interval 2-alternative forced-choice discrimination task. We argued that individuals’ ability to detect subtle acoustic differences without assigning categorical labels may not provide the most direct representation of the size of their auditory targets, and we hypothesized that categorical labeling consistency from an identification task would be associated with production variability measured as area of the ellipse. In contrast with this prediction, the present study found no association between our perceptual measure and area of the ellipse.

There are several possible interpretations for this unexpected null result. One possibility is that the relationship between perception ability and production variability is not as simple as posited. While the current thinking is that the magnitude of production variability for each speaker is constrained by the narrowness of their auditory targets in the representation, it may be that individual differences in production variability are dominated by differences in the speech-motor system, with the size of the auditory target playing a relatively minor role. That is, any observed production variability may be primarily reflective of motoric noise that arises during the execution of each sound. Another possibility is that the hypothesized relationship does exist and categorical labeling consistency is an appropriate measure, but limitations of our implementation of the identification task prevented us from capturing that relationship. For example, we synthesized the continua using model speakers’ natural productions instead of participants’ own speech. This choice was aligned with the methods used in both Perkell et al. (2008) and Franken et al. (2017). However, this potentially introduces a mismatch between the perceptual measure and the production variability measure, since participants are likely to make different perceptual judgments when hearing their own recorded speech versus model speakers’ speech. This point is bolstered by the findings reported in Chao et al. (2019), where there was a strong correlation between participants’ categorical perception boundary and a production-based categorical boundary for the same /ε/ - /æ/ contrast. In that study, participants’ own speech was used to create the synthesized vowel continuum for the identification task, specifically using participants’ median F1 and F2 values for each vowel as the two endpoints. Thus, it is possible that a relationship between categorical labeling consistency and production variability may be more consistently observed if self-produced speech is used in the identification task. However, it should be noted that the importance of using self-produced speech in the measurement of the perception-production relationship remains equivocal in the previous literature. For example, despite the aforementioned inconsistency, both Perkell et al. (2008) and Franken et al. (2017) observed significant associations between 4I2AFC discrimination ability and production variability where model speakers’ stimuli were used in the discrimination task. In addition, other studies have also reported that individuals’ perceptual ability, measured from a task that used model talkers’ speech stimuli, was related to the amount of adaptation to perturbed auditory feedback of their own production (Nault and Munhall, 2020). Interestingly, Schuerman et al. (2015) found that listeners were poorer at perceiving self-produced speech than a model talker’s speech in a spoken word recognition task, suggesting that there may be different processes involved in the perception of self-produced speech and that of other talkers (Schuerman et al., 2015). Of course, it is difficult to draw strong conclusions from results across these diverse studies using different perception and production tasks. Thus, further study is needed to directly explore whether the use of self-produced speech stimuli influences the ability to detect perception-production relations in a given task context, such as the present study of perceptual identification in relation to production variability.

To our knowledge, this is the first study directly examining the relationship between explicitly measured perception ability and the centering ratio measure of production variability. The centering process is thought to reflect a combination of the narrowness of the auditory target, production variability, and the auditory feedback control mechanism (Niziolek et al., 2013, 2015; Niziolek and Kiran, 2018; Bakst and Niziolek, 2019). There are a few possible reasons that may account for the lack of a significant association reported here. First, as mentioned in the hypothesis, it is possible that categorical labeling consistency is not an optimal measure to capture the auditory-perceptual processes involved in centering. Given that centering involves adjusting natural productions, which are likely to fall within the category for a speech sound, it depends on detecting subtle acoustic (or somatosensory) differences between the target and small deviations from that target. In this context, a perception task that taps into within-category discrimination ability might provide a better correlate of centering than the identification task used here.

In addition, similar to the points raised earlier, the lack of a relationship may be attributable to limitations of implementation in the present study. As stated previously, it is a drawback that the identification task did not use participants’ own recorded speech, since centering measures correction to self-produced speech. An additional limitation lies in the calculation of centering itself. As detailed above, the magnitude of centering is dependent on the amount of initial variability in a speaker’s productions. Even though we accounted for this by normalizing each participant’s centering to their initial variability, there still exists a potential ceiling effect on the centering ratio. That is, for participants who had a small amount of initial variability, the centering ratio does not necessarily reflect the participant’s true ability to perceive and respond to auditory feedback, because there was no need to correct their initial productions. The centering ratio for such participants would then represent a source noise in the correlation analyses, which could contribute to the lack of a significant association.

While centering has been discussed within the context of auditory feedback control, it is important to acknowledge that somatosensory feedback control might also be involved in the self-correction process, because both sensory domains are active in speech motor control. Niziolek et al. (2015) specifically examined the role of somatosensory feedback in self-correction and found that participants exhibited centering behavior even when their auditory feedback was masked by noise, although the amount of centering was less than when auditory feedback was available. Moreover, previous studies have suggested that individuals’ acuity might not be uniform across auditory and somatosensory domains (Fucci, 1972; Tremblay et al., 2003; Nasir and Ostry, 2008). In fact, in an implicit speech adaptation study where participants received simultaneous perturbation in both auditory and somatosensory feedback, Lametti et al. (2012) suggested that individuals tended to exhibit a “sensory preference,” i.e., a tendency to adapt more to the perturbation in their preferred sensory domain. This means that the lack of an association between categorical labeling consistency and centering ratio in the present study could be due to the fact that some participants might attend more to their somatosensory feedback than their auditory feedback. This, however, was not able to be examined in the present study because we did not measure participants’ somatosensory acuity. Future studies are needed to investigate whether there is an association between somatosensory acuity and measures of production variability.

In sum, the present paper started from the theoretical assumption that individuals with higher perceptual ability for a contrast can be expected to produce the same contrast with greater between-category separation and lower within-category variability. We directly tested this assumption by examining individual differences in perception and production of the American English /ε/–/æ/ contrast. In contrast with most previous research, this paper focused on measuring perception using categorical labeling consistency from an identification task, which was posited to bear a more direct relationship to the size of the auditory target than low-level measures of auditory discrimination. We examined whether individuals’ categorical labeling consistency was related to vowel contrast distance and to two measures related to production variability, namely area of the ellipse and centering ratio. The results of the study did not show significant associations between categorical labeling consistency and any of the production measures. These null results suggest that the relationship between perception and production may be more complicated than what has been posited in a widely adopted theoretical framing in terms of the size of the auditory targets of speech. However, it remains entirely possible that the theoretically predicted relationship is accurate, but our current measurement approach is not sensitive enough to capture it. Specifically, while we proposed to improve on previous perceptual measures by using a measure of categorical labeling consistency, we did not use participants’ own productions to create the synthesized vowel contrast continuum used in the identification task. Given that Chao et al. (2019) reported a strong relationship between the perception-based categorical boundary and the production-based boundary when self-produced speech was used to create the perceptual stimuli, it is of importance to investigate whether associations between categorical labeling consistency, vowel contrast distance, and the two measures of production variability might be observed if labeling consistency is measured using individual-specific acoustic continua. Furthermore, in the specific context of the centering ratio measure, it seems likely that a task measuring individuals’ ability to detect within-category differences may be more relevant for examining the relationship between perception and production variability. Taken together, the results of the current study suggest that follow-up research along the above lines is needed to further understand the nature of perception and production relationship.

## Data Availability Statement

All of the dataset and R scripts for reproducible statistical analysis and plots can be found here: https://osf.io/t7ry2/.

## Ethics Statement

The studies involving human participants were reviewed and approved by University Committee on Activities Involving Human Subjects, New York University. The patients/participants provided their written informed consent to participate in this study.

## Author Contributions

H-SC and TM developed the concept of the experiment, analyzed the data, and wrote the manuscript. H-SC, AB, and TM developed analysis plan and generated hypotheses. CN provided scripts and assistance for centering analysis. H-SC, CN, AB, and TM interpreted the results. CN and AB provided comments and suggestion to the manuscript. All authors contributed to the article and approved the submitted version.

## Conflict of Interest

The authors declare that the research was conducted in the absence of any commercial or financial relationships that could be construed as a potential conflict of interest.

## Footnotes

1 It is worth mentioning that different discrimination tasks were used between Perkell et al. (2004b) and Ghosh et al. (2010). The former used an ABX discrimination task, whereas the latter used a 4-interval 2-alternative forced-choice task with an adaptive staircase procedure. However, relative to perception of vowel contrasts, perceptual performance for consonant contrasts is thought to be less affected by task effects. That is, they tend to be perceived categorically across tasks (Pisoni, 1973). This could account for the similar results found by these two studies despite the use of different discrimination tasks.

2 Above it was noted that continuum steps oversampled in the probable boundary region, such that the middle five steps were closer in acoustic space than the outer six steps. Our data showed that all participants had a boundary region within the middle five steps of the continuum (mean = 5.75, SD = 0.7, min = 4.46, max = 6.96). This suggests that the non-uniform spacing does not pose a problematic confound for interpretation of the present results.

3 A boundary width of 0 was assigned by convention when the logistic regression did not converge and therefore a valid estimate of the slope was not available.
